# Informing iQOS Regulations in the United States: A Synthesis of What We Know

**DOI:** 10.1177/2158244019898823

**Published:** 2020-01-09

**Authors:** Carla J. Berg, Yael Bar-Zeev, Hagai Levine

**Affiliations:** 1 The George Washington University, Washington, DC, USA; 2 The Hebrew University of Jerusalem, Israel; 3 Hadassah Ein Kerem Medical Center, Jerusalem, Israel

**Keywords:** tobacco industry, tobacco control, marketing, alternative tobacco products

## Abstract

The tobacco industry offers various products, including heated tobacco products (HTPs). Philip Morris International’s (PMI) “iQOS” has the greatest HTP market share, as well as research on its use and impact. iQOS was released in 2014 and is now in more than 40 countries. The U.S. Food and Drug Administration announced permission for PMI to sell iQOS in the United States in April 2019, and iQOS was launched in October 2019. Decisions pending its modified risk tobacco product (MRTP) application will occur subsequently. The U.S. regulatory efforts regarding iQOS could be informed by examining (a) Philip Morris USA other product marketing efforts and (b) the iQOS market in countries where it is available. This article briefly addresses these two points with extant literature and suggests that future research should address important gaps in what is currently known, including strategic international collaborations and research, which historically has been critical for advancing tobacco control globally.

## Food and Drug Administration Tobacco Regulation

Tobacco use is the single largest preventable cause of disease and death in the United States (U.S. Department of Health and Human Services, 2014). Since 2009, the Food and Drug Administration (FDA) has regulated cigarettes, smokeless, and roll-your-own tobacco. The FDA finalized a rule, effective August 8, 2016, to regulate all tobacco products. This claimed the FDA’s regulatory authority over the manufacturing, marketing, and distribution of all tobacco products, including the broad range of alternative tobacco products, including e-cigarettes, cigars, and other products.

To stay in or enter the U.S. market, manufacturers of new tobacco products that are not substantially equivalent must submit a Premarket Tobacco Application (PMTA) to the FDA for any tobacco product (or modification of products) commercially marketed in the United States after February 15, 2007 (Tobacco Control Legal Consortium, 2017). Manufacturers can also submit applications for the *Modified Risk Tobacco* Product (MRTP) category, which would grant permission to use in marketing “modified risk statements” (i.e., claims that it reduces risk of tobacco-induced disease, has lower levels/is free of a harmful substance). To inform regulatory decisions, the applicant must provide data to the FDA to estimate the impact of these products and related marketing on public health. In addition, MRTP applications require an assessment of the impact of “modified risk statements” presented in marketing materials simulating how consumers encounter such messages in real life (Ashley, 2014, 2015).

## Heated Tobacco Products

**Figure 1.**
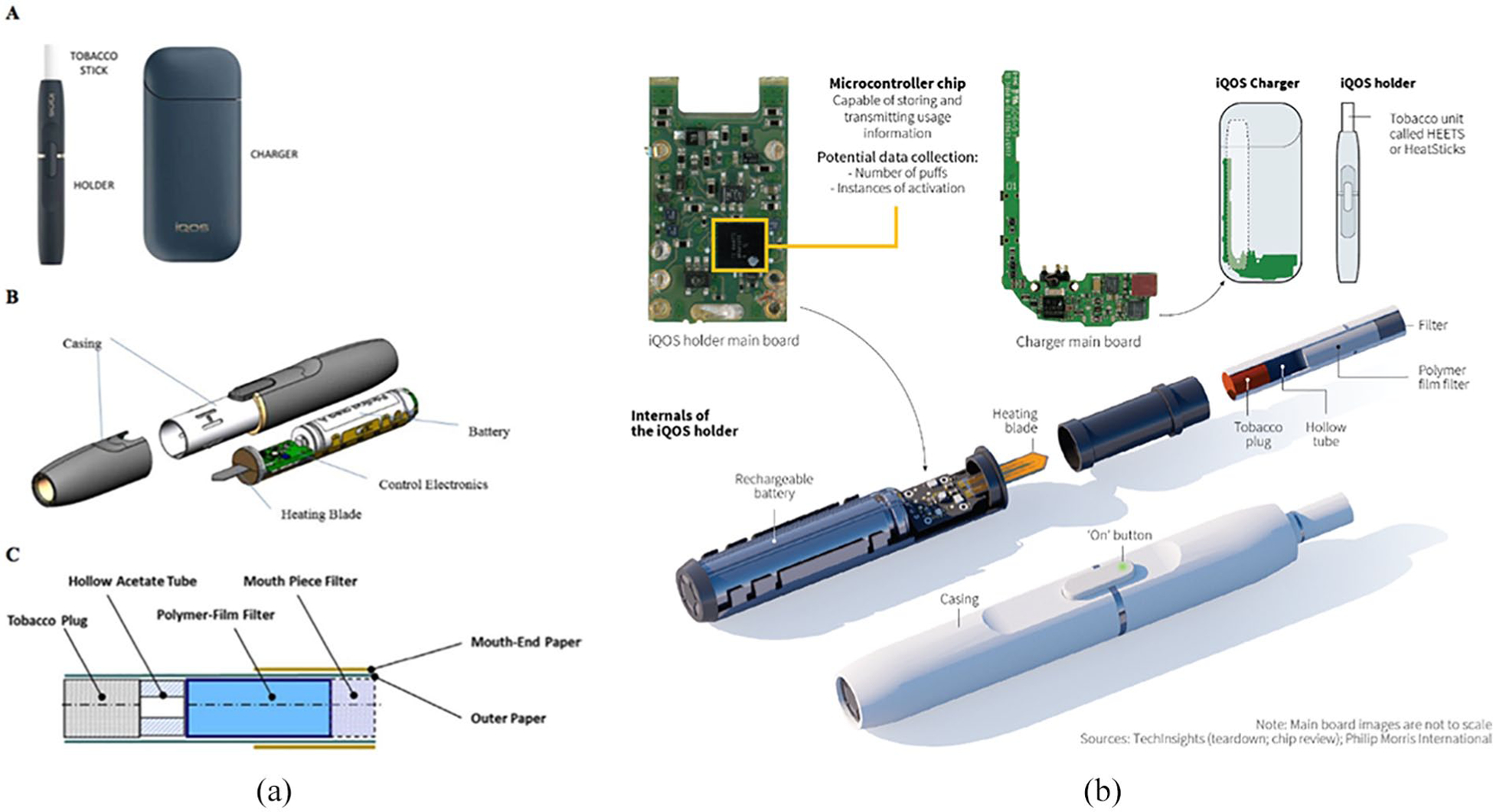
iQOS product diagrams (A) iQOS components and use and (B) iQOS data collection capacity. *Source*. https://tobaccocontrol.bmj.com/content/27/Suppl_1/s118; https://www.reuters.com/investigates/special-report/tobacco-iqos-device/ *Note*. Users push the tobacco unit into the iQOS holder, press a button to activate a battery-powered heater, and inhale the nicotine-containing vapor. The device heats disposable tobacco sticks (HEETS) soaked in propylene glycol at 350°C and produces an inhalable aerosol.

Heated tobacco products (HTPs) are electronic tobacco products that heat tobacco (Meckley et al., 2004; Stapleton et al., 1998) and include Philip Morris International’s [PMI] “iQOS,” British American Tobacco’s “Glo,” and Japan Tobacco International’s “TECH.” Interest in HTPs has dramatically increased, suggesting the potential of global expansion of HTPs (Caputi et al., 2017; Tabuchi et al., 2017). iQOS has the greatest share of the HTP market, as well as research on its use and impact (“Global Heat-Not-Burn Tobacco Products Market 2018–2022,” 2018; Trefis, 2017; Figure 1A; Auer et al., 2017; PMI, 2018). iQOS was released in Japan in 2014, is now in more than 40 countries (Liu et al., 2018, 2019; Tabuchi et al., 2016, 2017), and was launched in October 2019 in the United States, specifically in Atlanta and Georgia (FDA, 2019).

HTPs are marketed as less harmful products. A World Health Organization (WHO) report on HTPs highlighted that HTP marketing acknowledges the health risks of traditional cigarettes and indicates that HTPs are “cleaner,” “reduced-risk” products (WHO, 2018). Initially, most published research regarding iQOS were funded by PMI and its affiliates (Glantz, 2018a), indicating lower harmful tobacco carcinogen levels (Brossard et al., 2017; Jaccard et al., 2017; Ludicke et al., 2017, 2018; Martin Leroy et al., 2012; Pratte et al., 2017; Roethig et al., 2005, 2007). However, independent analyses of PMI’s research (Glantz, 2018a) elucidated that (a) PMI’s list of 58 harmful and potentially harmful constituents was lower in iQOS emissions compared with cigarette smoke (St. Helen et al., 2018); (b) PMI only reported on 40 of 93 constituents on the FDA’s list for iQOS (St. Helen et al., 2018); (c) 56 other constituents not included on either list were higher in iQOS emissions (St. Helen et al., 2018); (d) iQOS and conventional cigarettes showed no difference in most biomarkers of potential harm (Glantz, 2018b); and (e) iQOS has possible hepatotoxicity and potential for unexpected organ toxicity not previously associated with cigarettes (Chun et al., 2018). In addition, PMI’s guiding framework for its research, its Population Health Impact Model, excludes morbidity, underestimates mortality, excludes alternative tobacco products, does not include FDA-recommended impacts on nonusers, and underestimates impact on other population groups (Max et al., 2018).

Research independent from the industry has documented that the aerosols released by iQOS contain many of the same harmful or potentially harmful substances found in cigarettes (e.g., polycyclic aromatic hydrocarbons, carbon monoxide, formaldehyde cyanohydrin, *tobacco*-specific nitrosamines), as well as 84% of the nicotine found in cigarette smoke (Auer et al., 2017; Bekki et al., 2017; Davis et al., 2019; Leigh et al., 2018; St. Helen et al., 2018; Schaller et al., 2016). In addition, human (Moazed et al., 2018) and animal research (Nabavizadeh et al., 2018) showed no benefit of iQOS over cigarettes in relation to pulmonary function. Some investigators have proposed that PMI’s PMTA and MRTP applications for iQOS do not meet requirements for reduced harm or net public health benefit (Lempert & Glantz, 2018) and highlight the importance of research independent from the industry to estimate true public health impact (Glantz, 2018a). Research must address lingering concerns regarding the HTPs’ impacts on health, addiction, cessation of cigarette use among smokers, dual use of HTPs and cigarettes, and product use uptake by the nicotine naïve (e.g., nonsmokers, adolescents, young adults), among other concerns (Gottlieb & Zeller, 2017; Kim et al., 2018).

## Anticipating HTP Market in the United States

HTPs have been introduced into the U.S. market over the past 30 years (Dutra et al., 2017). The evolving tobacco market in the United States, which now includes a range of electronic nicotine delivery systems, among other products (Soneji et al., 2016; Strong et al., 2017), may provide a pivotal time for uptake of products like iQOS. Even as the FDA approved the PMTA for iQOS in April 2019 (FDA, 2019) and PMI is beginning to expand iQOS markets in the United States, PMI is also pursuing the MRTP categorization (FDA, 2018a, 2018b; National Public Radio, 2018). However, a federal advisory committee recommended FDA rejection of the MRTP application in January 2018 (Lavito, 2018). The FDA is not bound by the panel’s recommendations but often follows them.

To advance regulatory considerations of iQOS in the United States as its markets expand, it is critical to anticipate PMI’s iQOS marketing strategies (American Marketing Association, 2016; e.g., product design, placement, and pricing; marketing channels, messaging strategies, target markets) both as a tobacco product and as a product within the MRTP category (Henriksen, 2012; Lee & Kim, 2015). To do so, two major strategies could be leveraged. Research can examine (a) the marketing strategies previously used by Philip Morris USA (PM) to launch other emergent tobacco products such as e-cigarettes and (b) how PMI has marketed iQOS in countries where it is available.

## Prior Philip Morris USA Marketing Strategies

In examining marketing strategies previously used by PM to launch other emergent tobacco products in the United States, three products are highly relevant. First, Accord was an HTP released by PM in the late 1990s. iQOS’s design and marketing are similar to Accord’s (Elias et al., 2018). Aerosol chemistry data comparing iQOS and Accord indicated that iQOS reduces user exposure to some compounds but raises others (Elias et al., 2018). PM marketed Accord in several ways (e.g., less smoke, “enjoy everything you love about smoking”); particularly relevant, Accord was marketed as a “cleaner” tobacco product in an attempt to address smokers’ growing health concerns without making explicit health claims (Elias et al., 2018). However, PMI claims in its MRTP application that iQOS reduces health risk (Elias et al., 2018).

Another product is MarkTen, an e-cigarette launched by PM in 2013 and discontinued in 2018. One recent study (Haardörfer et al., 2017) examined differential advertising strategies used from 2013 to 2015 by four major U.S. e-cigarette brands: Njoy, Blu, Vuse, and MarkTen. These companies showed distinct spending trajectories overall and across media channels, with Njoy and Vuse spending a higher proportion of their dollars on TV, and Blu and MarkTen spending more on print. However, expenditures went down despite continued increases in social media activity (Berg et al., 2019; Haardörfer et al., 2017; Huang et al., 2014, 2018). In relation to marketing messages, key themes used by these four brands included switching from cigarettes, circumventing smoke-free policies, and technological advancement (Haardörfer et al., 2017). Specific to MarkTen, ads prominently featured young adults, females, and racial/ethnic minorities, as well as messaging regarding the technology and innovation of the product (Haardörfer et al., 2017). Other research identified noteworthy aspects of MarkTen marketing, such as messaging likening it to cigarettes (e.g., “delivers nicotine and provides a flavor and physical sensation similar to that of inhaled tobacco smoke”) but also using the term “vapor” prominently, distinguishing it from cigarettes and indicating reduced harm (e.g., “does not burn tobacco,” “does not generate or emit smoke”; Dutra et al., 2017). MarkTen ads also featured health warning labels, despite such warnings not being mandatory, perhaps in an effort to be perceived as protectors of consumer well-being (Richtel, 2014).

A more recent product that is noteworthy and relevant is JUUL, an e-cigarette originally developed by PAX Labs that represents over 75% of the e-cigarette market in the United States (Lavito, 2018). Altria, the parent company of PM, has taken a 35% stake of JUUL (Lavito, 2018). JUUL is designed as a slim, high-tech device that is charged through USB ports and uses nicotine cartridges, or “pods,” containing quick-delivery nicotine salts that come in a variety of flavors (Brown & Xing, 2015). The reported popularity of JUUL among youth (Willett et al., 2019) has been in part due to its ability to quickly deliver nicotine and its effects (Jackler & Ramamurthi, 2019), youth-friendly flavors such as Fruit Medley and Crème Brulee, its trendy design (called the “iPhone of e-cigarettes”; Radding, 2015), and JUUL’s discreet profile, facilitating use in places where it is banned (Ramamurthi et al., 2018). JUUL’s marketing strategies have involved youthful looking men and women using JUUL (which influences perceptions of who should use them), how discrete the device and its use are (which influences perceptions of how, when, and where to use them; Ramamurthi et al., 2018), and messaging that misleads about the risk of addiction (which has significant impact on use; Harty, 2015; Huang et al., 2018). JUUL is also highly discussed on social media platforms such as Twitter, Instagram, YouTube, and Reddit, which allows messaging that circumvents policy and has reach to youth populations (Huang et al., 2018).

In summary, the harm reduction claims used by PM to promote Accord, MarkTen, and JUUL are highly relevant to iQOS, particularly given the MRTP application. Other ways in which these products were marketed, including the marketing channels, messaging strategies (e.g., “cleaner,” technology), and target markets (e.g., young adults), may also be relevant, and perhaps contingent on iQOS’s product classification in the United States.

## International Research Regarding iQOS and Its Marketing

Research regarding tobacco products, their marketing, and their use in other countries has been critical in informing U.S. tobacco control efforts (Berg et al., 2018; Parascandola & Bloch, 2016) and is particularly critical when products—like iQOS—are highly distinct in terms of their design, distribution, and marketing. Despite PMI and PM being distinct entities, they have an agreement regarding sharing iQOS technology; thus, PMI’s marketing practices will likely inform PM’s. Below, we draw on some research regarding iQOS in previously established markets of Japan (2014), Italy (2014), Switzerland (2015), Israel (2016), Korea (2017), and Canada (2017; see Table 1 for tobacco control and use context; Auer et al., 2017).

### Product Design

iQOS is available in different colors and limited edition designs (PMI, 2018). Such design features may aim to fulfill the role cigarettes once held for consumers, for example, how brands such as Marlboro versus Virginia Slims reflected consumer characteristics such as ruggedness versus femininity (WHO, 2018). Moreover, such design features may also be intended to mimic characteristics of Apple products (e.g., iPhone, iPad) that are prominent consumer products globally, particularly likely given the parallel nature of the iQOS brand name. In addition, iQOS collects user data (Figure 1B; Lasseter et al., 2018). iQOS customers register their device, and software can extract information about a user’s behaviors (e.g., puffs, consumption; Lasseter et al., 2018), a design feature also inherent in JUUL (Daley, 2019). Despite this capability to obtain consumer information, PMI personnel claim that the data are not attached to consumers but rather the device and is only used to address technical problems (Lasseter et al., 2018). However, prior research has shown that the tobacco industry has used such databases in their marketing historically (Lewis & Ling, 2016). Thus, PMI’s use of this feature warrants investigation, particularly in regard to the use of this technology to learn about and influence consumers.

### Product Distribution

iQOS distribution is unique in that, in most markets, iQOS devices and accessories (e.g., cleaning sticks, leather wrap) can generally be purchased in only two ways—by purchasing them at iQOS specialty stores or by ordering the device online. The heat sticks—HEETS—are then purchased at traditional retailers (e.g., convenience stores, gas stations; PMI, 2018). Use of dedicated retail stores for various HTP brands is prominent (WHO, 2018). Also, the “bait and hook” pricing strategy is often used with HTPs; the base device (e.g., the iQOS device) is sold at a discounted price, with a recurrent price being charged for refills (e.g., HEETS; WHO, 2018). Research is needed to examine who is enticed by such distribution and pricing strategies and their impact on uptake and continued use.

### Marketing Communication Channels

PMI has used various approaches to promote iQOS. iQOS point-of-sale marketing is highly relevant but understudied. One recent study in Canada found that iQOS boutique stores use aggressive promotional activities, including deals involving exchanging cigarette packs or lighters for an iQOS device, social events, and membership programs, with signage reading “Building a Smoke-Free Future” and sales representatives regularly smoking iQOS (Mathers et al., 2018). Another study of HEETS retailers conducted in Israel (Bar-Zeev et al., 2019) indicated that the price for a HEETS package was an average 9.5% more expensive than cigarettes. While posted ads were uncommon, product displays were prominent. HEETS packages were often allocated particularly prominent placement and often within the line of vision for youth (Bar-Zeev et al., 2019; Halpern-Felsher, 2019).

Other approaches used to promote iQOS by launching media campaigns through both traditional and new media channels. In Israel, for example, iQOS penetration started in 2016 with unrestricted marketing and advertising (Kopel et al., 2017; Rosen & Kislev, 2018). PMI launched their iQOS campaign “Smoke-Free Israel” beginning with internet/social media and then full-page and large ads in print press (Rosen & Kislev, 2018), as well as press articles with prominent positive iQOS marketing content (Auer et al., 2017). PMI invested 22 million Shekels (~US$6 million) in 2017 on iQOS direct advertisement (60% in print, 40% digital; Linder-Ganz, 2018). Their marketing efforts involved community activators and brand ambassadors to promote iQOS through various channels, ranging from individual and community engagement to social media (PMI Digital Lab, 2018; WHO, 2018). The use of social media is noteworthy, given its low cost, high impact, and capacity to circumvent policy (Huang et al., 2014, 2018; Kavuluru et al., 2019).

### Public Relations Strategies

PMI has been cognizant of targeting policymakers (Bansal et al., 2019), press (Rosen & Kislev, 2018), and merchants (Bar-Zeev et al., 2019; Mathers et al., 2018). For example, PMI’s “Smoke-Free Israel” campaign targeted policy makers and the general public, emphasizing its alleged potential for harm reduction and the justification for regulation and policies different from traditional cigarettes (e.g., taxation, smoking and advertising bans, health warning labels; Rosen & Kislev, 2018), similar to its situation in Italy (Liu et al., 2019). The campaign included meetings with government officials and policymakers, as well as a public campaign targeted young people, which emphasized iQOS’s cleanliness (Rosen & Kislev, 2018). PMI’s campaign initially resulted in iQOS being exempt from tobacco regulations, which was later reversed, after fightback from a strong public health coalition (Kopel et al., 2017). Other data indicate that PMI aligns with press to promote their campaign messages (Rosen & Kislev, 2018) and with merchants by providing demonstrations and free samples (Bar-Zeev et al., 2019).

### Target Markets

Marketing campaigns, such as those developed by the tobacco industry (Anderson et al., 2005; Ling et al., 2004), are based on market research. Market research divides populations using segmentation based on some type of similarity, such as consumer sociodemographic profiles, behaviors, reactions to marketing messages, or psychographic characteristics (Ling et al., 2004; Ling & Glantz, 2002).

Considering sociodemographics and prior tobacco use behaviors, in the United States, from 2016 to 2017, adult awareness of HTP increased from 9.3% to 12.4%, ever use increased from 1.4% to 2.2%, and current use doubled from 0.5% to 1.1%, with greater use among racial/ethnic minorities and current smokers (Nyman et al., 2018). Research in countries with existing iQOS markets, specifically in Italy (Liu et al., 2019) and Korea (Kim et al., 2018), indicates that the number of never smokers who have already tried or intend to try iQOS is comparable to or greater than that of current smokers (Liu et al., 2019) and that current iQOS users were more likely to smoke conventional cigarettes and/or e-cigarettes (Kim et al., 2018). Collectively, these findings contradict the tobacco industry’s claims that conventional cigarette smokers will switch to HTP and may also highlight concerns regarding differential marketing on segments of the population (e.g., racial/ethnic or sexual minorities). However, to date, little non-industry-sponsored research has used market segmentation research to determine who is being targeted through different media channels or messaging strategies.

### Advertising Messaging Strategies

The psychographic segmentation approach distinguishes populations by individual attitudes, needs, wants, beliefs, goals, and lifestyles to develop targeted marketing messages and strategies (Katz & Lavack, 2002; Sepe et al., 2002). Health may be one important value held by consumers, and indeed, one prominent messaging strategy involves claims that iQOS is a reduced-risk product (despite contradictory evidence; Glantz, 2018a; WHO, 2018). How these types of messages are perceived must be examined. A critical review of PMI research submitted to the FDA regarding statements that “switching completely to iQOS reduces risk” indicated that current smokers did not understand the phrase “switching completely” and that iQOS users are not likely to “switch completely” (McKelvey et al., 2018). Another study found that “lower exposure” claims misled the public to perceive lower risk even though no lower risk claims were explicitly made (El-Toukhy et al., 2018).

Other messaging strategies have included a focus on other values and lifestyle characteristics. For example, iQOS has been marketed as a satisfactory alternative to traditional cigarettes, with some data supporting this assertion (Caputi, 2017; Tabuchi et al., 2016). However, users in Switzerland and Japan reported less satisfaction from HTPs versus cigarettes (Hair et al., 2018). As other examples of messaging themes, in Japan, iQOS is being marketed as a clean, chic, and pure product, which resonated well in Japan given the strong cultural values of order, cleanliness, quality, and respect for others (Hair et al., 2018). Indeed, Japanese iQOS users are reportedly motivated to use iQOS to socialize with nonsmokers (Hair et al., 2018). These are just a few examples of messaging strategies that could be employed in the United States.

## Conclusion

In summary, as iQOS markets in the United States begin to expand, regulatory efforts can be informed by the literature gleaned from prior “reduced harm” products marketed by PM in the United States as well as research of existing iQOS markets. First, the language and imagery used in advertising iQOS needs to be scrutinized to determine the extent to which consumers are interpreting or misinterpreting the product as less harmful, despite iQOS not currently being considered a modified risk product. Similarly, regulatory efforts must consider consumer perceptions of how and why the product should be used (e.g., to facilitate cessation of regular cigarettes, to project positive self-image) and who should use them (e.g., young people, current regular cigarette users) based on marketing materials. Relatedly, given iQOS software and the ability to collect user data (Lasseter et al., 2018), regulation is needed regarding the extent to which these data are being used by PMI to monitor and/or promote iQOS use among users. In addition, regulatory efforts must also consider PMI’s interactions with policymakers, press, and merchants; prior work has documented the relevance of doing so (Bar-Zeev et al., 2019; Mathers et al., 2018; Rosen & Kislev, 2018).

In addition, research must strategically build on this literature. Several key strategies could advance the literature and fill gaps critical to FDA regulation. First, strategic international collaborations are needed to advance and inform regulatory efforts for the broad range of emerging tobacco products. Such research is critical before and after the introduction of new products into various markets with differing regulatory environments to advance tobacco control globally and to anticipate how PMI will market iQOS in different regulatory contexts (O’Connor, 2012). Relatedly, research should compare responses to the iQOS product and its marketing channels and messaging across segments of populations in different countries to determine the extent to which similar consumer segments are being targeted in these countries, as well as the generalizability of findings across contexts. Particularly relevant to PMI’s MRTP application, research must examine consumer perceptions of harm reduction messaging in iQOS marketing. Countries with fewer advertising restrictions may be strategic settings to examine consumer reactions to actual ads in real-life settings. Finally, comprehensive case studies of iQOS marketing in countries where it is available are needed to provide in-depth information regarding the actual marketing strategies being used. Indeed, common themes across HTPs regarding their marketing and distribution channels have been identified but not fully studied within a single market (WHO, 2018). Israel, for example, could be one strategically chosen country. In Israel, iQOS has and will face regulatory changes over the course of a short 4-year period. Initially, in 2016 to 2017, iQOS was not designated as a nontobacco product and thus was not subject to regulatory oversight. Then, from 2017 to 2020, it was designated as a tobacco product (Rosen & Kislev, 2018) and subject to relatively weak legislation. In 2020, iQOS will face new tobacco control policies in Israel including increased restrictions on advertising and requirements on packaging (Baumer, 2019; Wootliff, 2018). Contexts such as this offer the opportunity to see how PMI will operate and adapt within different policy contexts within a single country over time.

Ultimately, several key themes regarding the marketing of novel tobacco products, particularly, iQOS, have emerged across contexts, tobacco products, and time. U.S. tobacco regulatory efforts, in general and specific to iQOS, could be advanced by considering what we know and by strategically building on the literature.

## Figures and Tables

**Table 1.** Tobacco Use and Policy Environment of Six Countries.

Characteristic	Japan^a^	ltaly^b^	Switzerland^c^	lsrael^d^	Korea^e^	United States^f^
Ratification of FCTC	2005	2008	N/A	2005	2005	N/A
Year iQOS launched	2014	2014	2015	2016	2017	2019
iQOS-classified tobacco	No	Yes	No	Yes	No	Yes
Smoking prevalence	%	%	%	%	%	%
Adult						
Males	33.7%	28.3%	26.9%	41.2%	42.1%	19.5%
Females	10.6%	19.7%	19.7%	19.3%	1.0%	15.0%
Children						
Males	1.6%	2.0%	2.1%	2.7%	2.5%	1.3%
Females	1.4%	2.6%	2.2%	1.2%	0.2%	1.2%
Smokeless Tobacco	N/A	0.6%	2.7%	N/A	N/A	2.2%
Policy						
HWLs						
Type of HWL	Text	Graphic	Graphic	Text	Text	Text
% of pack covered	30%	65%	43%	30%	n/a	50%
Plain packaging	No	No	No	No	No	No
iQOS included in packaging policies	No	*Text covering 30% of package	No*	Yes	Yes	Yes
Advertising bans						
Direct bans	N/A	National and international TV/radio; local magazines/newspapers; billboard/outdoor, point-of-sale and internet advertising	National and international TV/radio	National TV/radio	N/A	National TV/radio
Indirect bans	N/A	Appearance in TV/films	N/A	Appearance in TV/films	N/A	N/A
iQOS included in advertising ban	N/A	Yes	No	Yes	N/A	N/A
Smoke-free air						
Places covered by smoke-free air policy	None	None	None	Health care, educational, and government facilities	Health care and educational facilities; public transport	Government facilities
iQOS included in smoke-free policies	N/A	*Prohibited on school premises	N/A	Yes	Yes	Yes
Taxation						
% Retail price excise tax*	63.1%	57.9%	53.5%	68.4%	0.0%	37.8%
Tax on iQOS	See^a^	See^b^	See^c^	68.4%	See^e^	TBD

*Source*. https://tobaccoatlas.org/; https://www.tobaccocontrollaws.org/; https://globaltobaccocontrol.org/e-cigarette_policyscan; https://tobaccoatlas.org/; https://www.tobaccocontrollaws.org/; https://globaltobaccocontrol.org/e-cigarette_policyscan; https://untobaccocontrol.org/impldb/

*Note*. 1. No country has a comprehensive smoke-free air policy. 2. *Of 70% World Health Organization (WHO) Benchmark. 3. Recent and upcoming policy changes highlighted by the below references. FCTC = WHO Framework Convention on Tobacco Control; HWL = health warning labels.

TBD = To be determined.

a In Japan, a new tax category will be created for heat-not-burn tobacco products and the taxation system will be revised in consideration of the product characteristics. Heat-not-burn tobacco are controlled under the Tobacco Business Act and taxed under the Tobacco Tax Act and other relevant laws. *Source*. https://untobaccocontrol.org/impldb/wp-content/uploads/Japan_2018_report.pdf.

b Some taxes apply. *Source*. https://untobaccocontrol.org/impldb/wp-content/uploads/Italy_2018_report.pdf.

c Switzerland as not ratified the FCTC. Regulations that will regulate nicotine-containing e-cigarettes as tobacco products are under development. *Source*. https://globaltobaccocontrol.org/e-cigarette/switzerland.

d In Israel, IQOS declared not tobacco product May, 2015; IQOS declared tobacco product May, 2017; IQOS taxed as tobacco product January, 2018. New policy on advertising and packaging to be implemented in 2019. *Sources*. https://www.timesofisrael.com/knesset-stubs-out-ads-for-cigarettes-with-historic-life-saving-law/; https://www.calcalistech.com/ctech/articles/0,7340,L-3753353,00.html.

e Subject to a number of taxes and charges (national health promotion, tobacco consumption, local education, and individual consumption taxes) proportional to 1,799 won/mL (approx. US$1.53) nicotine liquid; in addition, there is a waste charge of 24 won/20 cartridges (approx. US$0.02) and a 10% value-added tax (VAT). *Source*. https://globaltobaccocontrol.org/e-cigarette/republic-korea.

f The United States signed the FCTC in 2004 but has not ratified it.
